# Gender differences in the association between grip strength and mortality in older adults: results from the KORA-age study

**DOI:** 10.1186/s12877-016-0381-4

**Published:** 2016-11-30

**Authors:** Marjan Arvandi, Barbara Strasser, Christa Meisinger, Konstantinos Volaklis, Raffaella Matteucci Gothe, Uwe Siebert, Karl-Heinz Ladwig, Eva Grill, Alexander Horsch, Michael Laxy, Annette Peters, Barbara Thorand

**Affiliations:** 1 Institute of Public Health, Medical Decision Making and Health Technology Assessment, UMIT, Hall in Tirol, Austria; 2 Division of Medical Biochemistry, Biocenter, Medical University Innsbruck, Innrain 80, A-6020 Innsbruck, Austria; 3 Institute of Epidemiology II, German Research Center for Environmental Health, Neuherberg, Germany; 4 Department of Prevention and Sports Medicine, TUM, Munich, Germany; 5 Institute for Medical Information Processing, Biometrics and Epidemiology (IBE), and German Center for Vertigo and Balance Disorders, Ludwig-Maximilians-University Munich, Munich, Germany; 6 Department of Computer Science, University of Tromsø, Tromsø, Norway; 7 Institute of Health Economics and Health Care Management, German Research Center for Environmental Health, Neuherberg, Germany

**Keywords:** Aged, Gender, Weakness, Survival, Physical activity

## Abstract

**Background:**

Reduced muscular strength in the old age is strongly related to activity impairment and mortality. However, studies evaluating the gender-specific association between muscularity and mortality among older adults are lacking. Thus, the objective of the present study was to examine gender differences in the association between muscular strength and mortality in a prospective population-based cohort study.

**Methods:**

Data used in this study derived from the Cooperative Health Research in the Region of Augsburg (KORA)-Age Study. The present analysis includes 1,066 individuals (mean age 76 ± 11 SD years) followed up over 3 years. Handgrip strength was measured using the Jamar Dynamometer. A Cox proportional hazard model was used to determine adjusted hazard ratios of mortality with 95% confidence intervals (95% CI) for handgrip strength. Potential confounders (i.e. age, nutritional status, number of prescribed drugs, diseases and level of physical activity) were pre-selected according to evidence-based information.

**Results:**

During the follow-up period, 56 men (11%) and 39 women (7%) died. Age-adjusted mortality rates per 1,000 person years (95% CI) were 77 (59–106), 24 (13–41) and 14 (7–30) for men and 57 (39–81), 14 (7–27) and 1 (0–19) for women for the first, second and third sex-specific tertile of muscular strength, respectively. Low handgrip strength was significantly associated with all-cause mortality among older men and women from the general population after controlling for significant confounders. Hazard ratios (95% CI) comparing the first and second tertile to the third tertle were 3.33 (1.53–7.22) and 1.42 (0.61-3.28), respectively. Respective hazard ratios (95% CI) for mortality were higher in women than in men ((5.23 (0.67–40.91) and 2.17 (0.27–17.68) versus 2.36 (0.97–5.75) and 0.97 (0.36–2.57)).

**Conclusions:**

Grip strength is inversely associated with mortality risk in older adults, and this association is independent of age, nutritional status, number of prescribed drugs, number of chronic diseases and level of physical activity. The association between muscular strength and all-cause mortality tended to be stronger in women. It seems to be particularly important for the weakest to enhance their levels of muscular strength in order to reduce the risk of dying early.

## Background

Ageing is typically associated with a progressive loss of skeletal muscle mass in combination with a concomitant increase in body fat mass [1]. The decline in skeletal muscle mass (defined as sarcopenia) occurs at a rate of 3–8% each decade after the age of 30 years and has been linked to increased morbidity and mortality in older people [2–4]. Recently, low absolute grip strength was the predominant predictor of clinical outcomes, whereas low lean mass was not a consistent predictor of mortality in the Foundation for the National Institutes of Health (FNIH) Sarcopenia Project [5].

In the last decade, evidence from epidemiological studies has shown that muscular strength is inversely associated with all-cause mortality in middle-aged and older individuals [6–10], even after adjustment for several confounders including levels of physical activity and cardiorespiratory fitness. Furthermore, similar associations have been reported in several clinical populations, suggesting that muscular strength protects against mortality not only in healthy but also in diseased individuals [11].

However, little is known about the level of muscular strength required to protect against the risk of death. In an observational study, similar risk reductions were reported in men with medium versus high muscular strength [9, 12], suggesting that high levels of muscular strength in comparison to moderate levels do not provide additional protection regarding all-cause mortality. In contrast, a meta-analysis by Cooper et al. [12] showed a linear mortality risk reduction with higher grip strength in community dwelling men and women of any age. A recent study observed regional and gender-specific associations of muscle strength with mortality in older people [13]. Risk of death was significantly lower only in women with high grip strength, but declined linearly with increasing grip strength in men [13]. Similarly, other studies found differences in the relationship between muscular strength and mortality between older men and women [7, 14]. Therefore, the purpose of our study was to examine the gender-specific impact of muscle strength on mortality in older adults.

## Methods

### Participants

Data used in this study were derived from the Cooperative Health Research in the Region of Augsburg (KORA)-Age Study conducted in 2008–2009. This study is a follow-up of all participants who took part in at least one of the four cross-sectional MONICA (Monitoring of trends and determinants in cardiovascular diseases)/KORA surveys performed between 1984 and 2001 in the city of Augsburg and the two adjacent counties. Altogether, 1,079 people aged 65 years and older took part in the KORA-Age Study. All participants were followed up for 3 years regarding mortality. Participants with incomplete data on mortality (outcome), maximum grip strength (predictor) or potential confounders were excluded. The remaining 1,066 (530 men, 536 women) individuals were on average 76 years old (SD = 11). The study was approved by the local authorities, and all subjects provided written informed consent.

### Grip strength

Handgrip strength was assessed using the Jamar dynamometer (Saehan Corporation). The Jamar dynamometer was placed in the participant’s dominant hand. Three trials were allowed with brief pauses. Participants were encouraged to exert their maximal grip strength in kilograms (kg). The maximum value of three handgrip measurements, divided into three groups using the gender-specific tertiles as the cut-off points, was used in the analyses.

### Mortality

All-cause mortality during the time interval from enrolment to the end of follow-up was the main outcome of the study. Deaths were ascertained by checking the vital status of all those sampled in the KORA-Age study through the population registries inside and outside the study area. Death certificates were obtained from local health authorities.

### Confounders

Potential confounders were chosen based on literature research and expert opinions. Sociodemographic characteristics of participants (including age at enrolment and gender), body mass index (BMI) (calculated as weight [kg]/height^2^ [m^2^]), lifestyle factors and nutritional status were collected during a standardized face-to-face or telephone interview, or during a personal examination. The risk of impaired nutritional status was assessed using a short version of the ‘Seniors in the Community: Risk Evaluation for Eating and Nutrition, Version II’ (SCREEN II) questionnaire [15], in which lower values correspond to a higher risk. In the present study, nutritional status was classified into three groups using tertile values as cut-off points. Leisure-time physical activity during summer and winter was assessed based on two separate questions with a four-level graded scale (0, <1, 1–2 and more than 2 h per week). The responses for summer and winter were combined to define one variable whereby a participant was considered active if he or she participated in sports in summer and in winter and for more than 1 h per week in at least one season. A participant was classified as inactive if he or she was less active during leisure time. As previously described [16, 17], use of medication and supplements was collected through a database-supported computer software (IDOM: Instrument for data based assessment of medication). Participants were asked to bring all product packages of ingested medications and supplements to the study centre. The enquiry period covered the last 7 days prior to the interview and participants’ mode of ingestion (regularly or irregularly, i.e. as needed), mode of prescription (prescribed, recommended by physician, self-medication), dosage and frequency of ingestion were collected for each preparation. For the present analysis, the variable ‘number of drugs’ includes only regularly consumed drugs prescribed by a physician. Dietary supplements and herbal products as well as homoeopathic preparations were excluded. Information on chronic diseases was collected through a self-administered questionnaire mailed to participants or a standardized telephone interview. Groups of chronic diseases were defined as suggested by Chaudhry [18]. For the present analysis, cancer, lung diseases, heart diseases and stroke were selected as potential confounders.

### Statistical analysis

Descriptive results for continuous potential confounders were presented as median and interquartile range (IQR) and for categorical variables as frequencies (%). Log-rank tests were performed to detect significant differences in the survival times by categories of potential confounders. Cox proportional hazards models with and without adjustment for significant confounders were used to examine the association between grip strength and all-cause mortality. Three different models were calculated: (1) basic model without adjustment for confounders; (2) model adjusted for age and gender; and (3) model adjusted for significant confounders identified in the total population based on the change-in-estimate criterion in ‘purposeful variable selection’ by Hosmer and Lemeshow [19, 20]. The selection occurred in three steps: initially, all variables with a significance level of <0.25 in the univariate analyses were included. In the second step, all variables with a p‐value higher than 0.10 or a relative change of less than 20% in other regression coefficients were eliminated in a stepwise manner. Finally, all variables that were not initially included because of a *p*-value ≥0.25 in the univariate analyses re-entered the model one by one, and the elimination criteria in the second step were proofed for them. The results are presented as hazard ratios (HRs) and 95% confidence intervals (95% CI) for all-cause mortality across gender-specific incremental thirds of muscular strength using the third tertile as the reference group. An adjusted Kaplan–Meier curve for the predictor in the final model was calculated and compared with the Kaplan–Meier curve before adjusting for the confounders. As sensitivity analysis, maximum grip strength values were included in the model as a continuous variable, and HRs per 1 kg increase in the maximum value of the grip strength measurements were calculated. Furthermore, effect modification by gender with respect to the effect of grip strength on mortality was tested by inclusion in the models of an interaction term between grip strength and gender. Age-adjusted mortality rates per 1,000 person years across incremental gender-specific thirds of muscular strength with 95% confidence intervals were computed for men and women. Data analysis was performed using the statistical software package SAS, version 9.4 (SAS Inc., Cary, NC, USA).

## Results

### Descriptive characteristics

Maximum grip strength and selected baseline characteristics of the 1,066 included individuals (530 men/536 women) stratified by gender are presented in Table 1. Maximum grip strength had a median (IQR) of 28 (6) kg in the first, 36 (2) kg in the second and 44 (8) kg in the third tertile in men, and 16 (6) kg, 22 (4) kg and 28 (4) kg in women. Overall, 34%, 34% and 32% of men and 36%, 41% and 23% of women belonged to the first, second and third tertiles respectively. Men were more active (*p*-values = 0.0046) and had better nutritional status (*p*-values <0.0001). Median age, BMI and number of prescribed medications consumed regularly as well as the prevalence of major chronic diseases were almost the same in men and women.

**Table 1:** Participant characteristics of the KORA-Age Study (2008 – 2011) stratified by gender (*n* = 1,066)

	Men (*n* = 530)	Women (*n* = 536)
Maximum grip strength, kg, median (IQR^a^)	35 (10)	22 (6)
Low^b^	28 (6)	16 (6)
Moderate	36 (2)	22 (1)
High	44 (8)	28 (4)
Age, years (median (IQR))	76 (11)	76 (11)
BMI, kg/m^2^ (median (IQR))	27.9 (5)	27.9 (6)
SCREEN II nutritional status (n (%))		
≤ 36 points	152 (29)	199 (37)
> 36; ≤41 points	188 (35)	213 (40)
> 41 points	190 (36)	124 (23)
Physical activity (n (%))		
Active	303 (57)	260 (48)
Inactive	227 (43)	276 (52)
Number of prescribed drugs (median (IQR))	3 (4)	3 (3)
Cancer (n (%))	27 (5)	16 (3)
Cardiovascular diseases (n (%))	198 (37)	180 (34)
Lung diseases (n (%))	60 (11)	54 (10)

^a^ *IQR* interquartile range. ^b^Low: men, ≤30 (kg); women, ≤18 (kg). Moderate: men, >30 (kg) and ≤38 (kg); women, >18 (kg) and ≤24 (kg). High: men, >38 (kg); women, >24 (kg)

### Association between grip strength and mortality

Table 2 presents the number of deaths by tertiles of maximum grip strength for both men and women. The mean grip strength (±SD) was 34.7 (±8.7) kg and 20.7 (±6.0) kg for men and women respectively. Over the follow-up time period (2008 – 2011), 95 participants died (56 men and 39 women). Age-adjusted mortality rates per 1,000 person years across incremental gender-specific thirds of muscular strength (95% CI) were 77 (59–106), 24 (13–41) and 14 (7–30) for men and 57 (39–81), 14 (7–27) and 1 (0–19) for women.

**Table 2:** Number of deaths and mortality rates in men and women by tertiles of grip strength during the follow-up time in the KORA-Age Study (2008 – 2011)

Men	Maximum grip strength ≤30 kg	Maximum grip strength >30– < 38 kg	Maximum grip strength >38 kg	Total
Alive (n (%))	144 (80%)	168 (93%)	162 (96%)	474 (89%)
Dead (n (%))	37 (20%)	12 (7%)	7 (4%)	56 (11%)
Total	181	180	169	530
Mortality rate^a^	77 (59–106)	24 (13–41)	14 (7–30)	39 (31–51)
Women	Maximum grip strength ≤18 kg	Maximum grip strength >18– < 24 kg	Maximum grip strength >24 kg	Total
Alive (n (%))	162 (85%)	210 (96%)	125 (99%)	497 (93%)
Dead (n (%))	29 (15%)	9 (4%)	1 (1%)	39 (7%)
Total	191	219	126	536
Mortality rate^a^	57 (39–81)	14 (7–27)	1 (0–19)	25 (19–35)

^a^per 1,000 person years (95% CI)

Table 3 summarizes the results of the Cox proportional hazards models for the association between tertiles of maximum grip strength and all-cause mortality, with and without adjustment for different combinations of confounders in the total study population and stratified by gender.

**Table 3:** Crude and adjusted hazard ratios of all-cause mortality for tertiles of maximum grip strength in the KORA-Age Study (2008 – 2011)

	Grip strength	Model 1	Model 2	Model 3
Men				
	Low	5.49 (2.45–12.3)	2.55 (1.04–6.22)	2.37 (0.97–5.75)
	Moderate	1.66 (0.65–4.21)	1.12 (0.43–2.91)	0.97 (0.36–2.57)
	High	1	1	1
Women				
	Low	20.96 (2.56–154)	7.24 (0.94–56)	5.23 (0.67–40.9)
	Moderate	5.17 (0.66–40.8)	2.87 (0.36–23)	2.17 (0.26–17.7)
	High	1	1	1
Total study population				
	Low	7.20 (3.45–14.99)	3.11 (1.42–6.85)	3.33 (1.53–7.22)
	Moderate	1.96 (0.87–4.43)	1.31 (0.57–3.01)	1.42 (0.61–3.28)
	High	1	1	1

Model 1: unadjusted. Model 2: adjusted for age (in the total study population, adjusted for both age and gender). Model 3: further adjusted for age, nutritional status: tertiles 1/2 vs. 3, physical inactivity, number of prescribed drugs, lung diseases, CVD, cancer (in the total study population, also adjusted for gender). Grip strength: low: men ≤30/women ≤18 (kg), moderate: men >30– ≤ 38/women >18– ≤ 24 (kg) and high: men >38/women >24 (kg)

**Fig. 1:**
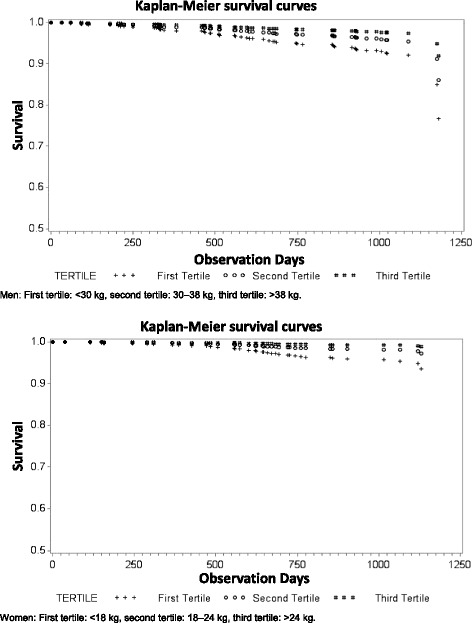
Adjusted Kaplan–Meier survival curves stratified by maximum grip strength tertiles for men and women in the KORA-Age Study (2008 – 2011)

After multivariable adjustment, HRs (95% CI) for all-cause mortality by tertiles of maximum grip strength were 5.23 (0.67–40.91) in women for the first tertile and 2.17 (0.27–17.68) for the second tertile versus the reference group (third tertile). In men, the respective HRs were 2.37 (0.97–5.75) and 0.97 (0.36–2.57). In a model combining data from men and women, multivariable adjusted HRs were 3.33 (1.53–7.22) and 1.42 (0.61–3.28) for the first and second tertiles respectively. The interaction between gender and grip strength tertiles was not statistically significant (*p*-value = 0.2174). Figure 1 shows the Kaplan–Meier survival curves according to tertiles of the maximum grip strength after adjustment for confounders in men and women.

## Discussion

The results of our study demonstrate that low handgrip strength is associated with all-cause mortality among older men and women from the general population after controlling for significant confounders, including age, nutritional status, number of prescribed drugs, diseases and level of physical activity. Further, we observed gender-specific differences with HRs being more than twice as high in women than in men. However, gender differences were not statistically significant, possibly because of the relatively small number of deaths especially in women.

Few studies have examined the effect of gender on the association between muscle strength and mortality with inconsistent findings [7, 10, 13, 14, 21, 22]. In three of them, muscle strength was not predictive of mortality in women [7, 14, 22], whereas three other studies [10, 13, 21] reported a significantly increased risk of mortality for women in the lowest quartile of grip strength. In the above studies, only three studies adjusted for physical activity [7, 21, 22]. We included level of physical activity, medical status and number of prescribed drugs, and we also accounted for nutritional status, all of which are independent predictors of mortality, in our final model. Although nutritional status was significantly associated with mortality (*p*-value = 0.01) and with grip strength (*p*-value = 0.0014), it was only a modest confounder of the association between grip strength and mortality. The HR in the full model without adjustment for nutritional status was 3.21 (1.48–6.98) for the first tertile versus the third tertile. Our results extend the limited literature by showing that, compared with men, it appears to be more important for older women to enhance their levels of muscular strength in order to reduce the risk of all-cause mortality [10, 13, 21]. The presence of chronic diseases commonly underlying death, nutritional status and physical activity did not explain the association, indicating that muscle strength may predict mortality through other mechanisms, such as factors secreted from the muscle cells, classified as myokines, which may protect against premature death [23].

In addition, little is known about the level of muscular strength required to protect against the risk of premature death. Data from the Aerobic Center Longitudinal Study, a prospective study of 8,762 men aged between 20 and 80 years, reported similar risk reductions in people with medium versus high muscular strength [9, 12], suggesting that having high levels in comparison with moderate levels of muscular strength does not provide additional protection. Indeed, in our study, men showed an elevated risk of mortality only when they had low levels of muscular strength (<30 kg), whereas women tended to show a higher risk of all-cause mortality with moderate muscular strength (between 18 and 24 kg) compared with those in the highest tertile of muscular strength. However, it has to be kept in mind that none of the individual HRs was statistically significant and, therefore, larger studies examining possible dose–response relationships and gender differences are urgently needed. The question arises whether the initial level or the rate of change in grip strength is more predictive of mortality. Danish data revealed significantly greater change for grip strength in men compared with women in later life, whereas the decline in grip strength was non-linear in women with a more pronounced decline with increasing age [24]. The data further suggested that the initial level of grip strength was more predictive of mortality than the rate of change, and the predictive effects were similar in men and women.

Our results suggest that older women are more sensitive to muscle strength alterations throughout ageing, probably as a result of sex differences in muscle mass as well as hormonal factors [25, 26]. It seems to be particularly important for the weakest to enhance their levels of muscular strength and build up muscle mass early in life, where targeted preventative efforts may be launched, to reduce their risk of premature death. On the other side, however, small improvements in strength levels (moving from the low to the middle tertile) may translate into great benefits in mortality risk reduction. Ultimately, randomized clinical trials with interventions aiming to increase muscular strength have to demonstrate whether increases in muscle strength at older ages indeed translate into reduced mortality rates.

The underlying mechanisms behind the benefits of muscular strength on mortality are still unclear but appear to be multifactorial. As there is a strong association between muscle strength and skeletal muscle mass, which is unquestionably a major tissue responsible for blood glucose disposal on account of its sheer size and ability to respond to insulin, the decline in muscle mass with age is linked to an increased risk of metabolic diseases such as type 2 diabetes associated with substantial premature death [27]. The findings of a recent study of 4,066 individuals, aged 20–85 years, revealed that, for every 0.05 decrement in grip strength (normalized as strength per body mass), there was a 1.26 times increased adjusted odds of diabetes in men and women [28]. The gender- and age-specific weakness thresholds to detect diabetes were lower in women than in men. In the past few years, skeletal muscle has been identified as a secretory organ producing and releasing several cytokines in response to contraction, which have been named myokines; these can influence the metabolism and function of muscle tissue and may counteract the harmful effects of proinflammatory adipokines [23]. Indeed, associations between chronic low-grade inflammation and sarcopenia are observed quite consistently, and inflammatory markers showed negative associations with both muscle mass [29] and strength [30, 31], as well as physical function [32]. Recent findings from the KORA-Age Study demonstrate higher concentrations of interleukin-6 and hs-CRP in older individuals with lower levels of muscular strength, independent of disease state, suggesting that the muscular system *per se* is effective in reducing low-grade inflammation [33]. Visceral fat has a high metabolic activity with deleterious effects on health contributing to muscle weakness and the frailty syndrome [34]. For example, the Baltimore Longitudinal Study of Aging has demonstrated in 786 individuals with a mean age of 66.3 years that adiposity is a significant predictor of lower muscle quality and strength [35]. The Toledo Study for Healthy Aging, a prospective study of 1,741 individuals aged ≥65 years, found a gender-specific nonlinear relationship between waist to hip ratio (WHR, a measure of central adiposity) and muscle strength [36]. In women, a normal BMI combined with either high or low WHRs were associated with a decrease or increase in strength, respectively. In contrast, men achieved their maximum strength at a WHR around 1 and the highest BMI. Thus, muscle strength may be determined by the relationship between WHR and BMI depending on gender. According to the Falls Risk and Osteoporosis Longitudinal Study in 171 men and women, aged 60–88 years old, better diet quality in females is associated with lower BMI and fat mass and higher lean mass, compared with males who appear to have better physical function, are less likely to self-report falls risk and have a better fat distribution, i.e. a lower android/gynoid ratio, which was significantly associated with better diet quality [37]. Moreover, low handgrip strength is a strong predictor of falls [38] and osteoporotic fractures [39], physical disability and frailty [40], which in turn have been found to be related to increased mortality risk [41]. It is, therefore, important for clinicians to be able to identify patients for whom low muscle strength is an important cause of their weakness because they are the most likely to benefit from therapies such as resistance training through enhanced muscle mass but also improved neural factors including motor unit recruitment and synchronization [42]. Thus, the assessment of grip strength could be a useful tool in clinical practice to identify older people with very low muscle strength at the greatest risk of all-cause mortality.

This study has several strengths and limitations. A major strength of this study was the prospective design, the inclusion of a number of participants aged ≥80 years and the use of an objective and standardized test for the assessment of maximal muscular strength. Limitations of the study are the relatively small sample size and short follow-up time. However, in old age, muscle atrophy can proceed dramatically, and even relatively small changes in muscle strength may have a significant impact on predictors of mortality such as glucose disposal, low-grade inflammation and osteoporotic fractures. Although we have adjusted for several confounding factors such as age, nutritional status, physical activity and chronic diseases, we were unable to adjust for cardiorespiratory fitness, which has been shown to be an independent predictor of all-cause mortality. Finally, as this is an observational study, we could only assess statistical associations and not causal relationships. Ultimately, randomized intervention studies aiming to increase muscular strength in older adults have to show whether the observed associations are causal and whether premature death can be prevented by increasing muscular strength.

## Conclusions

The findings of this study suggest that low muscle strength is inversely associated with mortality in old age, independent of potential confounders including age, nutritional status, number of prescribed drugs, chronic diseases and level of physical activity. This association tended to be stronger in women. Thus, the implementation of resistance exercise training for the weakest may be a promising means of reducing the adverse effects of low muscle strength on mortality and should be further tested in randomized clinical trials.

## Abbreviations

BMI: Body mass index
CVD: Cardiovascular disease
HR: Hazard ratio
hs-CRP: High-sensitivity C-reactive protein
IDOM: Instrument for data based assessment of medication
IQR: Interquartile range
KORA: Cooperative Health Research in the Region of Augsburg
MONICA: Monitoring of trends and determinants in cardiovascular diseases
SCREEN: Seniors in the Community: Risk Evaluation for Eating and Nutrition
WHR: Waist to hip ratio
